# Preparation of pH-Responsive Films from Polyvinyl Alcohol/Agar Containing Cochineal for Monitoring the Freshness of Pork

**DOI:** 10.3390/foods12122316

**Published:** 2023-06-08

**Authors:** Danfei Liu, Yunfei Zhong, Yumei Pu, Xiaoxuan Li, Siyuan Chen, Changfan Zhang

**Affiliations:** School of Packaging and Materials Engineering, Hunan University of Technology, Zhuzhou 412007, China; m18080502003@stu.hut.edu.cn (D.L.); yfzhong@hut.edu.cn (Y.Z.); m21085600005@stu.hut.edu.cn (Y.P.); m22077300009@stu.hut.edu.cn (X.L.); m20085600011@stu.hut.edu.cn (S.C.)

**Keywords:** pH-responsive films, cochineal, antimicrobial and antioxidant activities, pork freshness

## Abstract

This study reported the production of pH-responsive films based on 8 wt% polyvinyl alcohol solution/0.2 wt% agar solution incorporated with cochineal-loaded starch particles (CSN) (2, 4, 6 and 8 wt% on agar basis) by a casting process. Results revealed that CSN presented obvious color changes over the pH range of 2–12. FTIR, XRD spectra and SEM micrographs presented that the incorporation of CSN formed new hydrogen bonds with a matrix and a tighter network structure. A certain improvement was observed in the color stability, swelling index and functional properties (antimicrobial and antioxidant activities) but water solubility, water vapor permeability and water contact angle of the pH-responsive films were decreased by the addition of CSN. The release of cochineal was a rate-limiting step following the Korsmeyer-Peppas model. The agar/polyvinyl alcohol film containing 6% CSN (PVA/GG-6) exhibited the best sensitivity for ammonia detection and its limit of detection was 35.4 ppm (part per million) for ammonia. The application trials showed that the PVA/GG-6 film presented different color changes for pork freshness. Hence, these pH-responsive films can be used as potential packaging materials for tracking the freshness of protein-rich fresh food in a non-destructive way.

## 1. Introduction

As the demand for food safety increases, consumers are paying more attention to the quality of food. Packaging is a useful way of protecting food from the external environment (e.g., light, oxygen, bacteria) to guarantee food health and safety [1]. This has stimulated interest in the development of intelligent and active packaging materials with various functions such as antibacterial, antioxidant, shelf-life extension, food quality monitoring and so on [2]. Among smart packaging methods, pH-responsive indicators are an interesting and useful way to communicate with the product inside the package and convey information about product quality to the consumer without direct contact [3]. High protein foods (e.g., meat, seafood and dairy products) undergo changes in freshness during storage which result in the production of acidic or alkaline substances (e.g., ammonia, methylamine and dimethylamine) that can be detected by pH-responsive freshness indicators [4,5,6]. In general, pH-responsive freshness indicators are composed of two main parts, namely a polymeric carrier matrix and a pH-sensitive dye [7].

A large number of biodegradable natural polymers (e.g., sodium carboxymethyl cellulose, maize protein, starch, chitosan and k-carrageenan) have been used for the fixation of pH-sensitive dyes [8,9,10]. Agar is a natural polysaccharide material that is extracted from seaweed [9]. It is constituted by a backbone chain of β-D-galactose residues linked by alternating 1,3 glycosidic bonds and 3,6-endo-L-galactose residues and it has a complex multiphase structure [11]. Agar, owing to a significant number of surface hydroxyl groups, relies on hydrogen bonding and ionic interactions to form self-supporting large mesh-type hydrogels at room temperature [11]. Consequently, agar can be easily converted into a high-water content hydrogel suitable for gas protonation in the freshness indicator, thereby enhancing its gas response. However, agar films suffer from high brittleness and shrinkage, which restricts their application in packaging [9]. Blending agar with other polymers is an effective way to enhance the performance of agar-based films [12]. Polyvinyl alcohol (PVA) is a water-soluble polymer that is widely applied in packaging because of its chemical resistance, film-forming properties and high transparency [13]. Blending agar with PVA can improve the physical properties of a single film, resulting in better immobilization of pH-sensitive dyes [14].

Cochineal (CL) is a stable and safe natural pigment extracted from dried female insects, such as Dactylopius coccus [15]. These insects are parasitic on the cacti of the genus Cactus and feed on their sessile sclerotia [16]. Cochineal is highly soluble in water and polar organic solvents (e.g., acetone, ethanol and dimethyl sulfoxide) and exhibits colors ranging from orange to red and purple in terms of the pH values of the solution [15]. Cochineal is a natural colorant permitted by the FDA (Food and Drug Administration) for use in both food and pharmaceuticals and cosmetics [17]. Due to its noncarcinogenic nature and non-toxicity, excellent antioxidant properties and color-responsive properties, cochineal can be applied in the preparation of smart packaging [17]. To date, no research on cochineal-based smart packaging has been reported. The color response of cochineal to pH changes is affected by light degradation. The encapsulation of cochineal by nano- and submicron materials can enhance its stability and reasonably controlled release under suitable conditions [18]. Amylopectin particles exhibit excellent encapsulation effects on natural polyphenols, such as anthocyanins and catechins, and the phosphate group in the amylopectin particles interacts electrostatically with the hydrogen bonds in cochineal [19,20]. Meanwhile, the relationship between the color change of pH-responsive intelligent packaging systems and food freshness has been less studied. This study investigated the relationship between a pH-responsive intelligent packaging system and food freshness.

In the previous reports, a large number of natural active compounds (e.g., essential oils, natural extracts and their derivatives) have been used for the development of new active food packaging materials. Among the natural antioxidants/antimicrobials that have been used, essential oils (EOs), thyme oil, ZnO and anthocyanins have been extensively studied for use in active food packaging systems [21,22,23]. Meanwhile, active packaging based on cochineal has not been reported. Therefore, the application of cochineal to the preparation of active smart packaging can help promote the development of active smart packaging materials.

Since cochineal exhibits different colors at different pH values and studies on cochineal-based active smart packaging are not available, pH-responsive films based on polyvinyl alcohol/agar incorporated with cochineal-loaded starch particles were fabricated by the casting method. The effect of different cochineal-loaded starch particle contents on the color stability, water solubility, swelling property, water vapor permeability and water contact angle and functional properties of the films were considered. Additionally, the release kinetics of cochineal from the matrix through different models were researched. To test the application effect of the pH-responsive intelligent packaging film, it was applied to pork for monitoring the freshness changes during refrigeration.

## 2. Materials and Methods

### 2.1. Raw Materials

Fresh pork was obtained from a local market (Zhuzhou, China). Cochineal, amylopectin ((C_6_H_10_O_5_)_n_·xH_2_O, CAS No-9037-22-3) and Diammonium 2, 2’-azino-bis(3-ethylbenzothiazoline-6-sulfonate) (C_18_H_24_N_6_O_6_S_4_, CAS No-30931-67-0) were obtained by Macklin Biochemical Co., Ltd. (Shanghai, China). Polyvinyl alcohol 1799 ([-CH_2_CHOH-]_n_, degree of polymerization: 1750 ± 50, degree of alcoholysis: 98~99%, CAS No-9002-89-5), agar ((C_12_H_18_O_9_)n, density: 0.877, CAS No-9002-18-0), dimethyl sulfoxide (C_2_H_6_OS, purity ≥ 99%, CAS No-67-68-5), glycerol (C_3_H_8_O_3_, purity ≥ 99%, CAS No-56-81-5), ammonia hydrogen solution (25% in water) and acetic acid (HAc, 99.9%) were purchased from Shanghai Aladdin Biochemical Technology Co., Ltd. (Shanghai, China). *S. aureus* and *E. coli* were supplied by the Hunan University of Technology (Zhuzhou, China).

### 2.2. Preparation of Amylopectin and Cochineal-Loaded Starch Particles

Amylopectin particles were fabricated by using the previous method [24]. We mixed 0.3 g of amylopectin with 30 mL of dimethyl sulfoxide, and the mixed solution was then placed in boiling water and stirred for 1 h to fully gelatinize the amylopectin. The solution of gelatinized starch was slowly dropped into an absolute ethanol solution and stirred for 2 h. Lastly, amylopectin particles were washed four times with absolute ethanol and centrifuged at 6000 rpm for 3 min (Cence, TG16-WS, Changsha, China), after which they were dried in an oven (MEMMERT, UF55, Schwabach, Germany) at 40 °C for 12 h.

Additionally, 20, 30, 40 and 50 mg of cochineal were dissolved in an acetic acid buffer solution (pH = 2), and amylopectin particles were slowly added at a mass ratio of 1:10. The mixture was stirred for 3 h to obtain a cochineal-loaded starch particles suspension.

### 2.3. Characterization of Amylopectin and Cochineal-Loaded Starch Particles

#### 2.3.1. Encapsulation Efficiency (EE) of Cochineal

The suspension was centrifuged at 10,000 rpm for 30 min to obtain the composite precipitate. The absorbance of the supernatant was measured using a spectrophotometer (PerkinElmer, Lambda950, Waltham, MA, USA) at 496 nm. The free cochineal in the solution can be obtained from the standard curve (Figure S1): y = 25.33143x + 0.10189, R^2^ = 0.99958, where x (μg·mL^−1^) represents the concentration of cochineal and y represents the absorbance of the solution at 280 nm. The *EE* was obtained according to the equation:

(1) $$EE=1-\frac{C_{1}}{C_{2}}\times 100$$

where the *C*_1_ and *C*_2_ are the free cochineal concentration and total cochineal concentration, respectively.

#### 2.3.2. Morphological Structures and Particle Sizes of Amylopectin and Cochineal-Loaded Starch Particles

The particle sizes of the samples were tested according to the method of Teng et al. [25]. All samples were diluted with distilled water to a 0.01% solution. Particle size and Zeta potential measurement of samples were performed on a dynamic light scattering (DLS) analyzer (Article Sizing Systems Ltd., Nicomp Z3000, Port Richey, FL, USA). The samples were dispersed in distilled water (0.1%) and homogenized at a speed of 15,000 rpm for 3 min. One milliliter of the suspension was added to aluminum sheets and coated with gold spraying under a vacuum. The morphological structures of the nanoparticles were recorded by SEM (Carl Zeiss, Sigma 300, Oberkochen, Germany).

### 2.4. pH-Sensitive Property and Spectral Characterization of Cochineal Solutions

To analyze the pH-sensitivity property of cochineal, the 1.0 mg cochineal was dissolved in 50 mL of buffer solution (pH 2–12) to obtain the cochineal solution (0.02 mg/mL). The color information of the cochineal solution was observed and photographed. The absorption wavelength of the cochineal solution was characterized by an ultraviolet-visible spectrometer (PerkinElmer, Lambda950, Waltham, MA, USA) from 400 to 700 nm [14].

### 2.5. Fabrication of the Films

**Figure 1:**
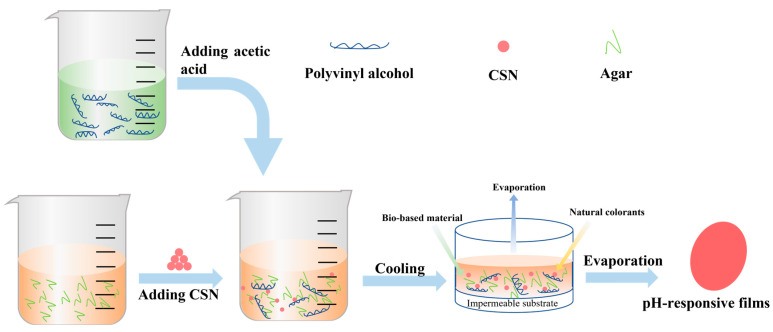
Schematic diagram of the preparation of the pH-responsive films.

The pH-responsive films were prepared by a modified method [10]. Polyvinyl alcohol (8 g) and agar (0.2 g) were dissolved in 100 mL of distilled water and stirred by a magnetic stirrer (LICHEN, DF-101S, China) at 90 °C for 3 h to obtain a polyvinyl alcohol solution and agar solution, respectively. The polyvinyl alcohol/agar solution was prepared by mixing a polyvinyl alcohol solution with an agar solution at a ratio of 2:1 *v*/*v* and stirring for 2 h at 60 °C. Glycerol was added to the mixed solution as a plasticizer (5% *w*/*w* mass of the mixed solution). When cooled to 70 °C, the polyvinyl alcohol/agar solution was added to the desired cochineal-loaded starch particles or cochineal powder (2%, 4%, 6%, 8% on agar basis) and continuously stirred to obtain the film-forming solution. Then, an appropriate amount of acetic acid is added to adjust the pH (2–3) of the film-forming solution. Lastly, 15 g of the solution was uniformly cast onto a glass Petri dish (diameter = 90 mm) and then dried in an oven (MEMMERT, UF55, Schwabach, Germany) at 45 °C for 48 h to obtain all films. The preparation schematic diagram was presented in Figure 1. On the basis of the content of the cochineal-loaded starch particles or cochineal powder (CL), the films were labeled as PVA/GG-2, PVA/GG-4, PVA/GG-6, PVA/GG-8, PVA/GG-2CL, PVA/GG-4CL, PVA/GG-6CL and PVA/GG-8CL. They were then vacuum sealed in aluminum plastic bags.

### 2.6. Structural Characterization of the Films

The microstructure of the film was recorded using SEM (Carl Zeiss, Sigma 300, Oberkochen, Germany). The samples were treated in liquid nitrogen for 10 min to obtain brittle fracture samples before the test. The truncated surface was glued to the conductive adhesive, and the sample was obtained by spraying gold for 45 s. The acceleration voltage during SEM shooting was 3 kV. The FT-IR analysis of the films was captured by an FT-IR spectrometer (Thermo Fisher Scientific, NicoletIS/10, Waltham, MA, USA) from 1000 to 4000 cm^−1^. X-ray diffraction of all samples was obtained using an X-ray diffractometer (Bruker, Bruker D2 PHASER, Karlsruhe, Germany) with the scanning range of 5–90° (2θ) under Ni-filtered Cu-Kα radiation.

### 2.7. Physical Characterization of the Films

#### 2.7.1. Optical Properties of the Films

The color information of the film was recorded using a colorimeter (X-rite, X-rite 530, Chicago, IL, USA) [10]. The total color difference (ΔE*) of the film was evaluated in accordance with the following formula.

(2) $$∆E^{*}={\left[{\left(L-L^{*}\right)}^{2}+{\left(a-a^{*}\right)}^{2}+{\left(b-b^{*}\right)}^{2}\right]}^{0.5}$$

where L* (91.72), a* (0.61), b* (−0.25) is the color values of the standard whiteboard.

#### 2.7.2. Swelling Index (SI) and Water Solubility (WS)

The test sample (60 mm × 60 mm) was pretreated in an oven at 70 °C for 12 h to obtain the initial mass *m* (g) of the sample [14]. After that, the samples were immersed in 50 mL of deionized water for 12 h. The excess water on the surface was then removed to obtain the mass *m*_1_ (g) at this point. Finally, the samples were dried in an oven at 70 °C for 12 h to obtain the sample mass *m*_2_ (g). The test was repeated 3 times for each sample. The swelling index (SI) and water solubility (WS) were calculated by the following formula.

(3) $$\mathrm{SI}\left(\%\right)=\frac{m_{1}-m}{m}\times 100$$

(4) $$\mathrm{WS}\left(\%\right)=\frac{m-m_{2}}{m}\times 100$$

#### 2.7.3. Water Vapor Permeability (WVP)

The film samples (5 cm × 5 cm) were placed on centrifuge tubes containing 40 g of anhydrous silica gel and subsequently stored in a desiccator with distilled water (100% RH). The weight of the film samples was measured every 24 h for 7 days until stabilization [12]. The *WVP* (×10^−11^ g m^−1^ s^−1^ Pa^−1^) of all films was obtained by the following equation.

(5) $$WVP=\frac{W\times x}{t\times A\times ∆P}$$

where *x*, *A*, Δ*P* and W/t represent the sample thickness (mm), water vapor permeation area of film surface (m^2^), mass variation of the silicone with time (g/s) and the differential vapor pressure inside and outside the film (Pa), respectively.

#### 2.7.4. Water Contact Angle (WCA)

The contact angle measuring instrument (Dataphysics, Dataphysics OCA20, Stuttgart, Germany) was utilized to determine the water contact angle on the surface of the film [9]. The film sample (40 mm × 40 mm) under test was affixed onto the horizontal moving stage of the contact angle measuring instrument. Then, 5 μL of deionized water was dropped onto the film surface and the WCA was measured on both sides of the water droplet. The WCA of each film was determined by calculating the average value from three measurements.

### 2.8. Functional Characterization of the Films

#### 2.8.1. Antioxidant Activities

The antioxidation activities were recorded using the former method [26]. Briefly, the sample (10 mg) was mixed with a solution of Diammonium 2, 2’-azino-bis(3-ethylbenzothiazoline-6-sulfonate) (10 mL, 6 mM) at room temperature for 30 min in the dark. The absorbance of the solution was measured at 734 nm using a spectrophotometer (Lambda950, PerkinElmer, Inc., Waltham, MA, USA). The antioxidation activity could be calculated by the following formula.

(6) $$Antioxidant\;activity\left(\%\right)=\frac{A_{c}-A_{s}}{A_{c}}\times 100$$

where the *A_c_* is the initial absorbance of the ABTS solution and *A_s_* is the absorbance of the mixed solution.

#### 2.8.2. Antibacterial Activities

The antibacterial properties of the pH-responsive film were measured by the previous method with some modifications [27]. Before testing, two bacterial cultures were properly diluted and counted on the agar medium to obtain a bacterial concentration of 10^6^ CFU/mL. All films were sterilized under an ultraviolet irradiation lamp for 30 min. The film sample (0.7 g) was mixed with 50 mL of bacterial solution (10^6^ CFU/mL) and incubated for 24 h using an oscillator (37 °C, 200 rpm) (Shanghai Yiheng Technology Co., LTD, THZ-100B, Shanghai, China). A PVA/GG film medium was used as the control group. The *OD*_600_ (absorbance at 600 nm) of the supernatant was measured by a spectrophotometer every 4 h for 24 h to monitor bacterial growth. The antibacterial efficiency (*AE*) was obtained as follows:

(7) $$AE\left(\%\right)=\frac{1-OD_{600}^{S}}{OD_{600}^{C}}\times 100$$

where the *OD*_600_ of sample medium ($OD_{600}^{S}$) and control ($OD_{600}^{C}$) is given.

### 2.9. Release Behavior

The release behavior of cochineal from pH-responsive films was measured in buffer solutions at 25 °C [27]. The films (0.5 g) were immersed in a 150 mL flask containing 100 mL of buffer solution and then placed in an oscillator (25 °C, 120 rpm). The absorbance was measured by regularly sampling at 280 nm using a spectrophotometer. The amount of cochineal released was determined from the standard curve (Figure S1): y = 25.33143x + 0.10189, R^2^ = 0.99958, where x (μg·mL^−1^) was the concentration of cochineal and y was the absorbance of the solution at 280 nm. The cumulative release was estimated using the following formula.

(8) $$\mathrm{Cumulative}\;\mathrm{release}\left(\%\right)=\frac{M_{t}}{M_{0}}\times 100\%$$

where *M_t_* (mg) is the released mass of cochineal at time *t* (min) and *M*_0_ (mg) is the total incorporated mass of cochineal in the film.

Ritger and Peppas’ formula was used to simulate the release of cochineal from the pH-responsive film (Ritger & Peppas, 1987) [28].

(9) $$\frac{M_{t}}{M_{\infty }}=K\times t^{n}$$

where *M_t_* (mg) is the released mass of cochineal at time *t* (min), *M_∞_*(mg) is the released mass of cochineal at equilibrium, *K* is the release rate constant and *n* is the release index. The models of first-order ($1-\frac{M_{t}}{M_{\infty }}=e^{-kt}$), second-order ($\frac{M_{t}}{M_{\infty }}=kt/\left(1+kt\right)$) are also presented.

### 2.10. Color Stability of the Films

To simulate a real storage environment, the films were stored under a D65 light source (color temperature: 6500 K) for 14 days. The related information (L, a, b values) of the samples was recorded by a colorimeter (X-rite 530, Chicago, IL, USA) every 2 days. The ΔE* value of the films was obtained by Equation (2).

### 2.11. Sensitivity to Ammonia of the Films

The sensitivity of the samples to ammonia was characterized by the previous method [10]. The trimmed film (2 cm × 3 cm) was fixed on the top of a sealed glass Petri dish. The desired gas concentration was achieved by injecting a certain volume of ammonia solution (0.3 M) into the Petri dish (90 mm). The color changes and parameters (R, G, B values) of the films were obtained using an iPhone 11 and colorimeter (X-rite 530, Chicago, IL, USA) under standard illuminant D65. The ultraviolet spectra of the films were measured by ultraviolet-visible spectrometry (Lambda950, PerkinElmer, Inc., Waltham, MA, USA). Ammonia concentration (*C*) in the test chamber and the sensitivity (*S*) were estimated using the following equation [29].

(10) $$C_{ppm}=\frac{V_{\mu L}\times D_{mg/L}\times W}{M_{g/mol}\times V_{L}}\times 22.4\times 10^{7}$$

where $V_{\mu L}$ is the volume of the ammonia hydrogen solution, $D_{mg/L}$ is the density of the ammonia hydrogen solution, W is the mass fraction of the ammonia hydrogen solution, $M_{g/mol}$ is the molar mass of the ammonia hydrogen solution and $V_{L}$ is the volume of Petri dishes.

(11) $$S\left(\%\right)=\frac{\left(R-R_{0}\right)+\left(G-G_{0}\right)+\left(B-B_{0}\right)}{R_{0}+G_{0}+B_{0}}\times 100$$

where the *R*, *G*, *B* are the final color parameters of the sample, and *R*_0_, *G*_0_, *B*_0_ are the initial color parameters of the sample.

### 2.12. Application of the Films

The pH-responsive films were applied as indicators to monitor pork freshness. Twenty grams of pork were placed in separate Petri dishes (90 mm diameter). The films were cut to a size of 10 mm × 20 mm and fixed in the top space of the Petri dishes, which were then placed in a 4 °C refrigerator for 7 days. The pH-responsive film images were captured daily using a camera, and Adobe Photoshop 2021 was used to obtain the related color information to calculate the corresponding ΔE* values. The pH and TVB-N values of pork were obtained with the method reported earlier [30]. The total viable microbial count (TVC) of the pork was tested using the previous method [9].

### 2.13. Statistical Analysis

The physical and functional properties of the films were tested at least three times. The results were shown as means ± standard deviation (SD). The data to be analyzed were statistically analyzed by one-way analysis of variance (ANOVA) in SPSS 23.0 software. Significant differences were detected by Duncan’s multirange analysis. *p* < 0.05 means the difference is statistically significant. Additionally, in accordance with the empirical equation previously reported, testing was conducted to ensure mean equality (EA%) or inequality (IA%) [27].

## 3. Results and Discussion

### 3.1. Characterization of Particles

**Figure 2:**
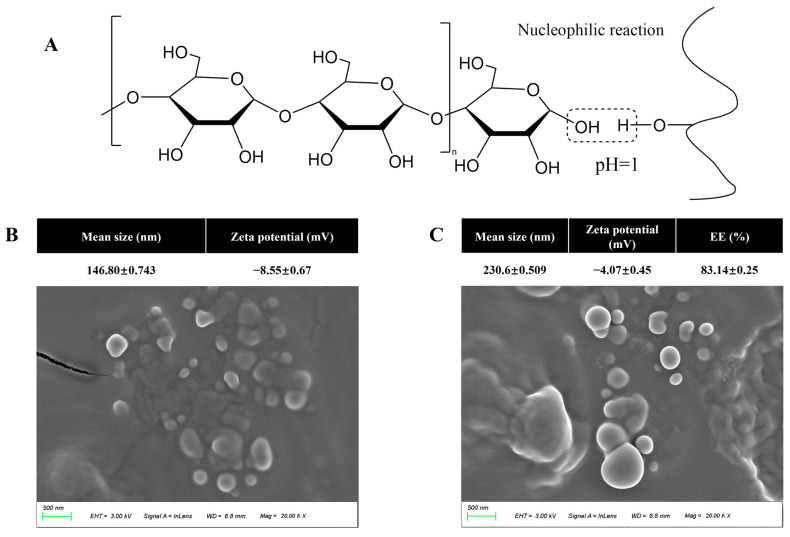
Encapsulation mechanism between cochineal and amylopectin particles (**A**), the SEM image, DLS and EE of amylopectin particles (**B**) and CSN (**C**).

As shown in Figure 2B, amylopectin particles exhibited an average particle size of 146.8 nm and an elliptical shape. After complexation with cochineal, the cochineal-loaded starch particles increased significantly to 230.6 nm due to the encapsulation of cochineal by amylopectin particles and adhesion to each other (Figure 2C), and overlap was observed. Furthermore, the negative zeta potential of amylopectin particles indicated that the particles have a small negative charge on the surface, which may be due to the phosphate group in the molecule of starch [31]. However, since the low potential indicates that the particles are less stable, they are more prone to agglomeration. In our study, the encapsulation efficiency of amylopectin particles for cochineal reached 83.14 ± 0.25%, indicating its preponderance in encapsulating cochineal. Additionally, it was also found that the positive charge of the carbon positive ion (as shown in Figure 2A, located in the hemiacetal hydroxyl group of amylopectin particles) was enhanced under acidic conditions. The nucleophilic reaction between starch particles and cochineal was improved. Qiu et al. also found that the nucleophilic reaction between starch nanoparticles and phenolic compounds was greatly affected by pH value [31].

### 3.2. Color and UV-Vis Spectra of Cochineal Solutions in Different Buffer Solutions

**Figure 3:**
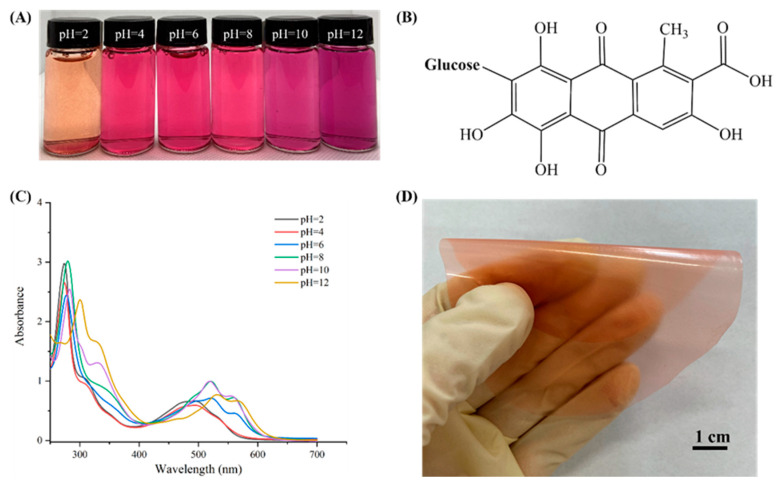
Color variation (**A**), structure (**B**), UV-vis spectra (**C**) of cochineal in different pH solutions (from 2 to 12), and pH-responsive films that are easily foldable (**D**).

The cochineal solution exhibited color changes in pH ranging from 2 to 12 (Figure 3A). Cochineal belongs to the anthraquinone pigment, with its structure mainly consisting of α-hydroxyanthraquinone in an acidic environment (Figure 3B), and its color is mainly orange and mauve [32]. At pH = 2, the cochineal solution appeared orange and the UV-vis spectrum showed the maximum absorption peaks (λ_max_) at 285 nm and 520 nm. With an increase in pH, the absorbance at 285 nm gradually decreased and the color of the cochineal solution turned from orange to red (pH = 2–6) (Figure 3A,C). Since the molecular structure of cochineal is an electron-donating absorbing system composed of carbonyl and hydroxyl groups, the hydroxyl groups at the α positions can be used as electron-donating substituents to produce the dark color effect [33]. At pH 8–12, the carbonyl and hydroxyl groups in the cochineal molecular structure would ionize, causing a change in their absorption peak and color [33]. Meanwhile, the maximum absorption peak (λ_max_) of the cochineal solution presented changes in a red shift from 279 nm to 300 nm. Additionally, the absorbance of the cochineal solution gradually decreased at both 279 nm and 517 nm, causing the color of the solution to change from red to purple. The results showed that cochineal can be used as a pH indicator to detect pH changes in food. Figure 3D shows the appearance of the pH-responsive film.

### 3.3. Swelling Properties

The swelling properties of films are important physical properties for their application in food packaging. As shown in Table 1, the WS value of the PVA/GG film was 38.76 ± 1.57. With the addition of CSN, the WS value of the pH-responsive film showed a trend of decreasing (*p* < 0.05). The main reason for the decrease of WS with the increase of CSN content may be related to the poor water solubility of CSN. Previous studies have reported similar behavior in the water-solubility of pH-responsive films; WS values decreased significantly with the addition of water-insoluble solids in the research [9]. In contrast, the WS value of the pH-responsive films increased with the addition of water-soluble extracts [10]. Due to the high WS value of the pH-responsive film, this film is not suitable for packaging water-based foods. SI values indicate the effect of moisture on the color response efficiency of pH-responsive films during storage. A high SI value indicates a faster dye release rate, which is not conducive to the color response efficiency of the pH-responsive films [10]. The SI values of all films are presented in Table 1. The SI value of the pH-responsive film increased and then decreased with increasing cochineal content (*p* < 0.05). The SI value is related to the total number of water molecules in the network structure of the film [10]. The increase in SI value was due to the lower content of cochineal with fewer intermolecular forces with the film-forming matrix, resulting in more contact points with water. After, the decrease in SI value was mainly due to the increased intermolecular interaction between the cochineal and the film-forming matrix, which is more compact in structure. The results showed that PVA/GG-6 and PVA/GG-8 films can be used for monitoring the freshness of food.

### 3.4. Water Vapor Permeability and Water Contact Angle

The water vapor permeability (WVP) of a packaging film is indicative of its ability to act as a barrier against water vapor and other volatile substances [34]. Table 1 showed that the PVA/GG film exhibited the highest WVP values (13.27 × 10^−11^ g m^−1^ s^−1^ Pa^−1^), while a significant difference in WVP was observed in the PVA/GG-2 film (12.83 × 10^−11^ g m^−1^ s^−1^ Pa^−1^). No changes were detected in the WVP of the PVA/GG-4, PVA/GG-6 and PVA/GG-8 films. The addition of CSN resulted in a reduction of the WVP value for the pH-responsive film. The formation of new hydrogen bonds between CSN and substrates results in a reduction of free hydroxyl groups and an increase in film crystallinity, thereby decreasing the WVP value of the film. On the contrary, while the cochineal rich in hydroxyl groups exhibited an increase within the film, only a limited number of free hydrophilic groups were provided by cochineal encapsulated in starch particles, resulting in a relatively minor enhancement of the water barrier capacity of the film. Several studies have demonstrated similar findings that the physical interaction between natural extracts and film-forming matrix molecules serves as a bridge between polysaccharide chains, leading to a compact structure that reduces the water vapor permeability of the packaging film [35,36].

The water contact angle (WCA) of all film surfaces was measured to further determine the surface hydrophobicity and wettability of the films. It can be utilized to characterize the wettability properties of a sample, including hydrophilicity (<90°), hydrophobicity (>90°), and superhydrophobicity (>180°) [37]. The WCA results of different films were presented in Table 1; the WCA value decreased significantly (*p* < 0.05) from 59.70° in PVA/GG film to 58.33°, 57.96°, 57.81° and 57.39° in the PVA/GG-2, PVA/GG-4, PVA/GG-6 and PVA/GG-8 films, respectively. The reduction in WCA indicated an enhancement of the hydrophilic properties on the film surface due to the incorporation of CSN. This was primarily attributed to the abundance of hydrophilic groups, such as carboxylic and phosphoric acid groups, within CSN, which resulted in increased hydrophilicity of the pH-responsive films [19]. However, Huang et al. discovered that the incorporation of arnebia euchroma root extracts into the AGAR film resulted in a transition from hydrophilic to hydrophobic properties [9]. The results indicate that the hydrophobic nature of the filler can significantly impact the wetting behavior of the pH-responsive film.

### 3.5. Morphology and Structure of the Films

#### 3.5.1. Scanning Electron Microscopy (SEM)

**Figure 4:**
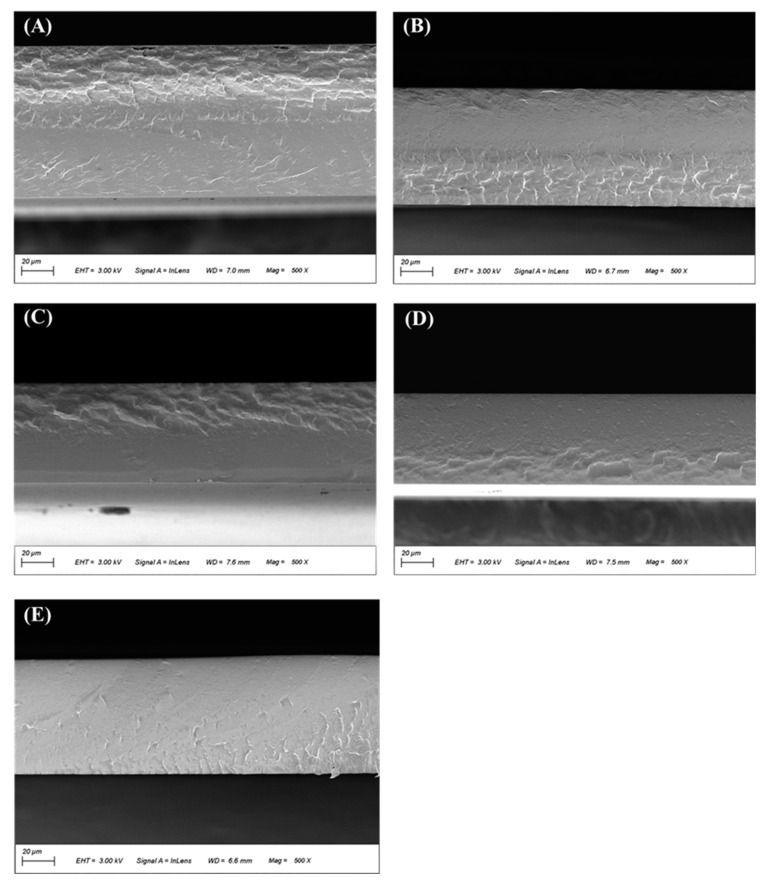
SEM micrographs on the cross-sections of (**A**) PVA/GG, (**B**) PVA/GG−2, (**C**) PVA/GG−4, (**D**) PVA/GG−6, (**E**) PVA/GG−8 films.

The microstructural information of the films obtained by SEM can be used to explain the changes in the macroscopic properties of the samples. The cross-sectional images of the films were indicated in Figure 4A–E. For the control film (Figure 4A), the cross-section was rough and cracked due to the relatively low compatibility between polyvinyl alcohol and agar. The films, after incorporating cochineal-loaded starch particles, presented a flat and smooth cross-section, indicating that cochineal-loaded starch particles were compatible with other polymers and thus improved the compatibility of polyvinyl alcohol and agar (Figure 4B). Furthermore, as the content of cochineal-loaded starch particles in films increased, the flatness of cross-sections increased (Figure 4C–E), suggesting that the cochineal-loaded starch particles were uniformly dispersed in polymers of the matrix and further interacted with it through intermolecular forces [38]. CSN molecules promoted intermolecular interactions between the polyvinyl alcohol and agar, leading to an increase in film crystallinity. Analogously, some research showed that the addition of poplar anthocyanin led to a more uniform continuous structure of the methylcellulose/chitosan nanofiber film [4]. In contrast, Ma and Wang (2016) [4] and Liu et al. (2021) [39] found that the cross-sections of colorimetric films based on a high content of grape skin anthocyanin and red cabbage anthocyanin became rough and cracked.

#### 3.5.2. X-ray Diffraction Spectra (XRD)

**Figure 5:**
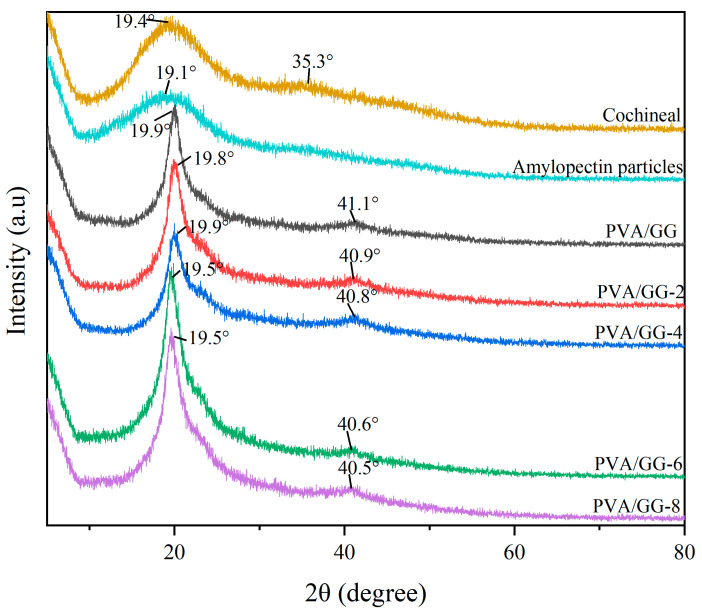
XRD patterns of the powders and PVA/GG films with different cochineal-loaded starch particle contents.

The crystallinity can reflect the compatibility and interaction between polymers [40]. Figure 5 shows the XRD patterns of the powder and the polyvinyl alcohol/agar films with and without cochineal-loaded starch particles. The cochineal and amylopectin particles presented broad amorphous peaks at 19.4° and 19.1°, indicating that they were amorphous structures, which was similar to the results of other researchers [41]. The crystallinity index of pure cochineal and amylopectin particles was 14.3% and 12.1%, respectively. The polyvinyl alcohol/agar film indicated a semi-crystalline character through a strong diffraction peak of 19.9° and a weak diffraction peak of 41.1°. The crystallinity index of the PVA/GG film was 27.8%, whereas the crystallization characteristics of the polyvinyl alcohol/agar films did not alter after the addition of cochineal-loaded starch particles, which was probably due to the low content of cochineal-loaded starch particles in films. Furthermore, as the content of cochineal-loaded starch particles in the film increased, the crystallinity index of PVA/GG-2, PVA/GG-4, PVA/GG-6 and PVA/GG-8 were 28.9%, 29.1%, 39.9% and 40.3%, respectively. This is due to the intermolecular hydrogen bonding between cochineal-loaded starch particles and the film-forming matrix after the addition of cochineal-loaded starch particles, which contributes to the orientation of molecules. FTIR analysis also further verified the existence of intermolecular hydrogen bonds between CSN and the film-forming matrix, whereas other studies have shown that a clear decreasing trend appeared in the peak intensity of starch/polyvinyl alcohol films with increasing betalains content in films [12]. Hence, the crystallinity of polyvinyl alcohol-based films is influenced by the content and composition of the polyphenols in the extracts.

#### 3.5.3. FTIR Analysis

**Figure 6:**
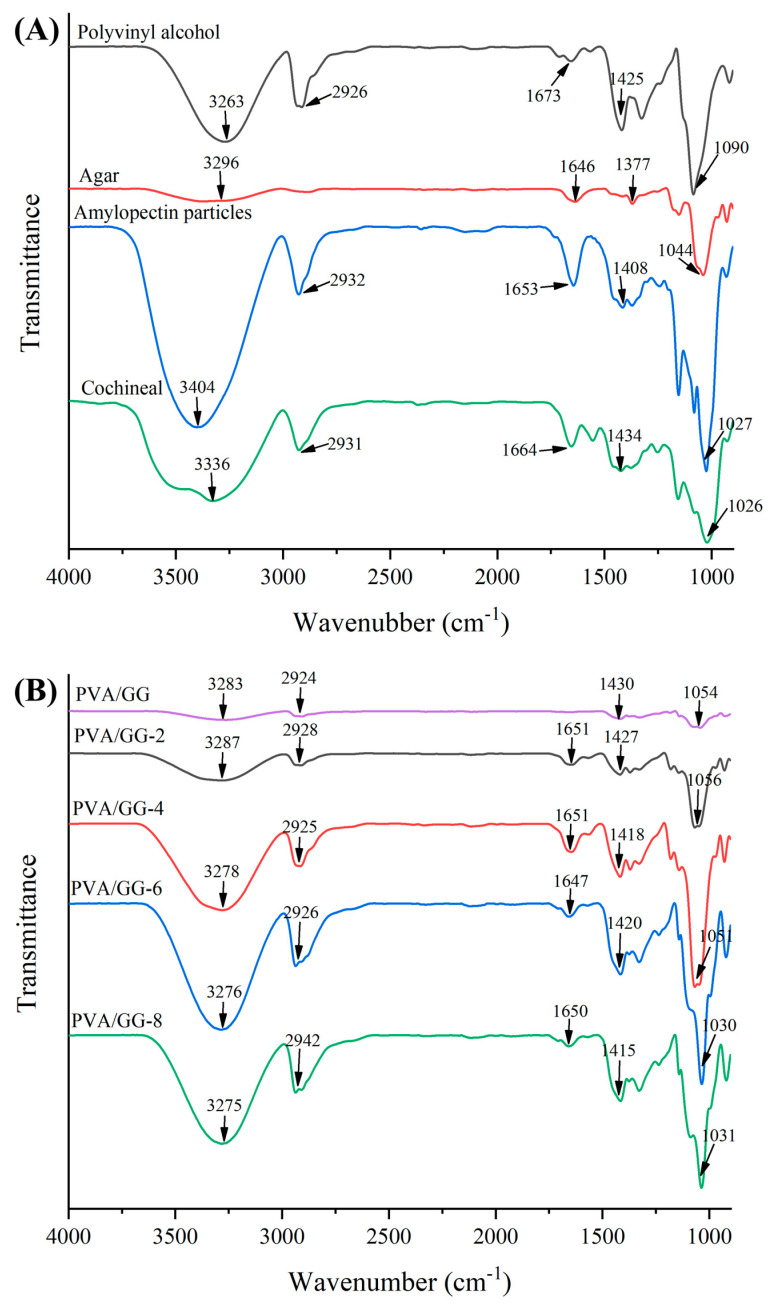
FTIR spectrum of (**A**) cochineal, amylopectin particles, polyvinyl alcohol, agar and (**B**) PVA/GG, PVA/GG−2, PVA/GG−4, PVA/GG−6 and PVA/GG−8 films.

FTIR spectra can be applied to detect the interaction of the molecules in the polymers (Figure 6A,B) [42]. The cochineal showed an absorption peak at 3336 cm^−1^ corresponding to O-H stretching. The characteristic absorption peaks of cochineal at 1664 cm^−1^ and 1434 cm^−1^ represented the alkene bonds in the aromatic ring structure composed of phenol and carbonyl groups, as well as the absorption peaks at 1026 cm^−1^, which were attributed to the C-H bending vibration of the aromatic ring [41]. The distinct peaks at 2931 cm^−1^, 1664 cm^−1^, 1434 cm^−1^ and 1026 cm^−1^ corresponded to C-H, C=O, C=C and C-O stretching of cochineal, respectively [41]. Amylopectin particles showed distinct peaks at 2932 cm^−1^, 1653 cm^−1^, 1408 cm^−1^ and 1027 cm^−1^ corresponding to C-H stretching vibration, O-H bending of bound water, C-H bending vibration and O-H stretching vibration in glucose units, respectively. In the spectrum of agar, peaks at 1646 cm^−1^, 1377 cm^−1^ and 1044 cm^−1^ were attributed to a C–N group, ester sulfate group and aliphatic ether group, respectively [43]. Polyvinyl alcohol presented two characteristic peaks at 1425 cm^−1^ and 1090 cm^−1^, corresponding to the bending of CH-CH_2_ and stretching of C-O in the basic carbon skeleton, respectively. Strangely, negligible shifts were exhibited in the stretching vibration of the O-H band after the incorporation of cochineal-loaded starch particles, suggesting that intermolecular hydrogen bonds formed between cochineal-loaded starch particles and the polymer matrix. With the addition of cochineal-loaded starch particles at 2%, 4%, 6% and 8%, the stretching vibrations of C=O bonds appeared at 1650 cm^−1^, 1649 cm^−1^, 1648 cm^−1^ and 1647 cm^−1^. Hence, FTIR spectra of PVA/GG films with cochineal-loaded starch particles indicated that the particles were well-dispersed in the polymer matrix.

### 3.6. Color Parameters and Stability of the Films

The color parameters and stability of the films were shown in Table 1 and Figure S2. The color of the polyvinyl alcohol/agar film was colorless, while the color of the pH-responsive films became darker with increasing content of cochineal-loaded starch particles. With the increase of cochineal-loaded starch particle content in the film, the *L* value of the film decreased, while the *a* and *b* values increased, indicating that the pH-responsive films became redder and yellower. This was because the more cochineal in the film, the darker the orange color of the film, the L value decreased and the a value and b value increased correspondingly.

The color stability of the pH-responsive films was characterized by measuring the color difference values (ΔE*) of all films stored under identical lighting conditions. The storage conditions of the films are critical for their application. As shown in Figure S2, the ΔE* values of the films increased with longer storage times when stored under scattered light due to cochineal decomposition or oxidation caused by ultraviolet light [16]. Furthermore, compared to polyvinyl alcohol/agar films, pH-responsive films exhibit better color stability due to the encapsulation protection of cochineal by amylopectin particles. The presence of amylopectin particles in pH-responsive films reduced the exposure of cochineal to ultraviolet light and slowed down the decomposition of the pigment.

### 3.7. Release Behavior Analysis of the Films

**Figure 7:**
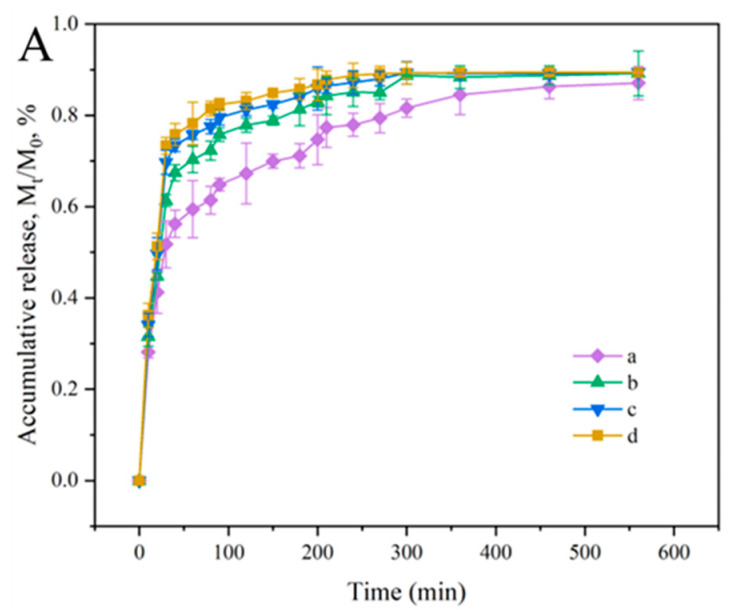

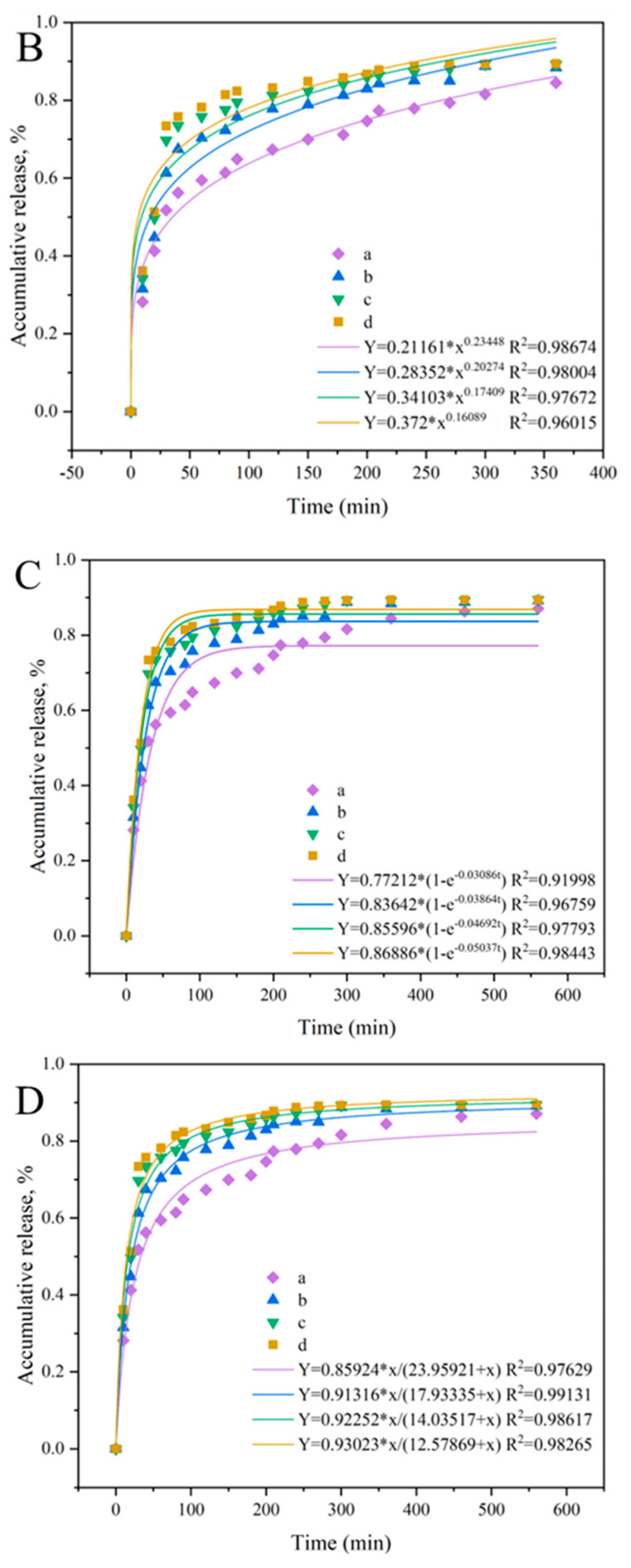
Release percentage (**A**) of PVA/GG−2 (a), PVA/GG−4 (b), PVA/GG−6 (c), PVA/GG−8 (d) films in deionized water based on models of Korsmeyer–Peppas (**B**), first-order (**C**), second-order (**D**).

In Figure 7A, the release of cochineal from the film in deionized water showed a controlled release behavior over time: an explosive release occurred in the first 20 min, followed by a gradual increase and then a calm period. The initial burst release was related to cochineal on the film surface; after which, the increase in release rate was mainly attributed to the need for more time for the cochineal trapped by water molecules to be released. Nonetheless, the cochineal content directly affected the diffusion driving force of cochineal, and the release rate increased with the increase of cochineal content in the film. The results were similar to previous work [44].

To further investigate the release behavior of cochineal in the films, three different typical models were applied to fit and analyze its release behavior (Figure 7B–D, insert: release equation and correlation coefficient R^2^). The Korsmeyer–Peppas model was used to simulate the release behavior of cochineal from the film at M_t_/M_0_ ≤ 2/3. The high value of R^2^ (0.96015–0.98674) confirmed that the Korsmeyer–Peppas model could be effectively employed to analyze the release behavior of cochineal. The release index n (0.16089–0.23448) showed that cochineal release followed Fick diffusion [45]. Meanwhile, comparisons showed that the release behavior of cochineal was consistent with both the second-order model (R^2^ > 0.97629) and first-order kinetic equation (R^2^ > 0.91998), suggesting that the diffusion mechanism of cochineal was a rate-limiting step.

### 3.8. Functional Properties of the Films

#### 3.8.1. Antibacterial Activities

All films were characterized for their antibacterial activities against *E. coli* and *S. aureus*. The control group showed three typical dynamic phase variations during bacterial growth (Figure S3A,C). During the lag phase, the bacterial population grew slowly due to adaptation to the new external environment [46]. Upon contact with the film, it exhibited a high antibacterial rate because the cochineal released from the film destroyed bacterial cell structures and killed some of them. In the exponential phase, the antibacterial rate was reduced due to excessive bacterial multiplication and the release of less cochineal to inhibit bacterial multiplication. The antibacterial rate gradually increased as bacterial growth entered a plateau and bacterial reproduction was limited by the massive release of cochineal and the lack of nutrients. Of note, the higher cochineal content in the films exhibited higher antibacterial activities due to the high cochineal content in the film leading to high release of cochineal from the culture medium, thus inhibiting bacterial growth more significantly.

The growth and antibacterial curves of *S. aureus* were similar to those of *E. coli* (Figure S3C,D). The results showed that the pH-responsive films presented an excellent antibacterial effect on two kinds of bacteria. Furthermore, the pH-responsive films exhibited significantly better antibacterial activity against *S. aureus* than *E. coli*, primarily due to differences in their cell membranes (*S. aureus* has a peptidoglycan layer as its outer layer while *E. coli* has a phospholipid membrane) [47].

#### 3.8.2. Antioxidant Activities

**Figure 8:**
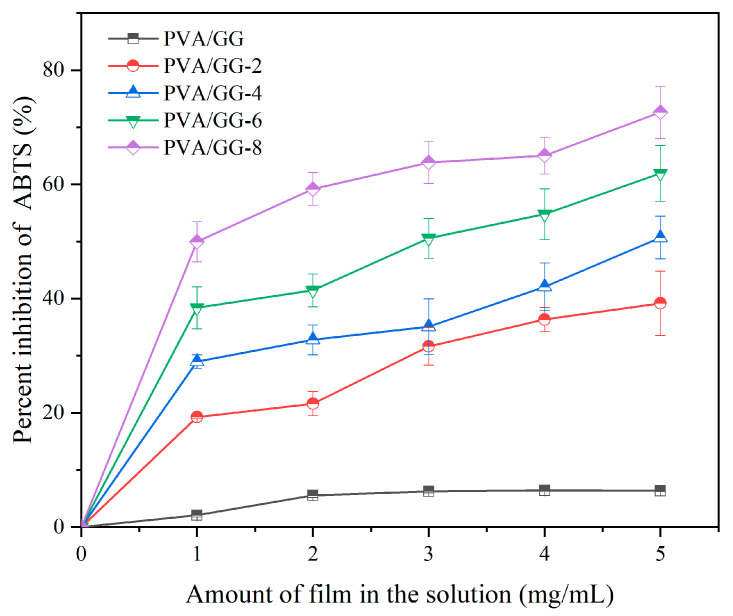
Antioxidant activities of the films.

Spoilage and nutrition loss of food are related to free radicals [48]. Cochineal exhibits a variety of biological activities. Furthermore, research on the reaction of cochineal with 2,2′-diphenyl-1-picrylhydrazyl (DPPH) and 2,2′-azinobis (3-ethylbenzothiozoline-6-sulfonate) radical cations (ABTS^+∙^) showed that cochineal possessed the ability to scavenge free radicals [49]. In this work, we tested the free radical scavenging ability of cochineal and pH-responsive films, as shown in Figure 8A. After accepting hydrogen atoms, ABTS^+∙^ became stable ABTS while cochineal was converted into a semi-quinone free radical stable structure [49]. Since the oxidation resistance of the PVA/GG film was low, the oxidation resistance of the pH-responsive films mainly derived from the action of cochineal. Furthermore, the content of cochineal in the pH-responsive films affected their oxidation resistance due to a larger release driving force, resulting in higher cochineal content in the solution. Currently, other studies found that the addition of anthocyanins, betalains and curcumins could enhance the antioxidant activities of packaged films. However, fewer studies have focused on the application of cochineal in preparing active packaging to prolong the shelf life of foods. Hence, this study is beneficial for promoting the application of a new natural dye (cochineal) in active packaging systems to protect packaged food from free radical-induced oxidation.

#### 3.8.3. Sensitivity of the pH-Responsive Films to Ammonia

**Figure 9:**
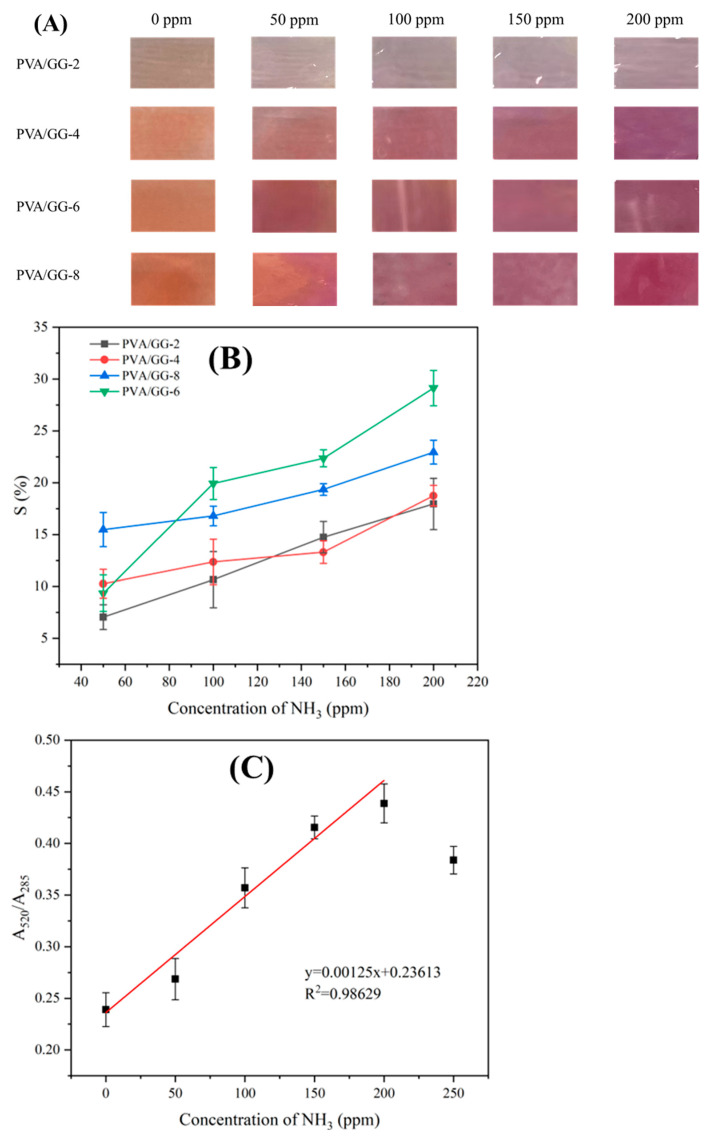
The color (**A**) and S (**B**) values of the pH-responsive films at different NH3 concentrations, as well as the relationship (**C**) between A520/A285 of the UV-vis spectrum and NH3 concentration variations.

Basic nitrogen compounds produced by the degradation of proteins and lipids will volatilize during changes in meat freshness, which can be used as an index to measure meat freshness. When the gas concentration of ammonia increased to 100 ppm, all films changed color from orange to purple (Figure 9A). Accordingly, the S value of the pH-responsive film to NH_3_ also increased as the concentration of ammonia vapor increased (Figure 9B). Meanwhile, the S value of the pH-responsive film showed an increase and then a decrease with the increase of cochineal content in the film, which might be due to the fact that the high cochineal content resulted in a decrease in the proportion of cochineal that underwent a color change. The results presented that the gas sensitivity of the film was greatly affected by the amount of cochineal in it. Qin et al. [12] found that the freshness indicators with high dye content exhibited more noticeable color changes to the naked eye.

The PVA/GG-6 film with the highest gas sensitivity was selected to determine its detection limit for NH_3_. Figure 9C showed that the absorption peak of the PVA/GG-6 film rose at 520 nm and fell at 285 nm, which was the same as the change of cochineal in the pH buffer solution. Through fitting A_520_/A_285_ with ammonia vapor, it was found that there was a linear relationship between A_520_/A_285_ and ammonia vapor, with a correlation coefficient (R^2^) of 0.98629 (Figure 9C). Hence, the limits of detection (*LOD*) for ammonia vapor in the PVA/GG-6 film were calculated as 39.4 ppm using the following formula. The results indicated that the PVA/GG-6 film could be used to detect the freshness of aquatic products and meat (35 ppm) in food packaging [29].

(12) $$LOD=\frac{3S}{N}$$

where *S* was the standard deviation (0.016401) of the blank group and *N* was the slope of the fitted curve.

### 3.9. Effectiveness of the Films to Monitor the Freshness of Pork

#### 3.9.1. pH, TVB-N and TVC Contents

In Table 2, the pH value of fresh pork was 5.11, while the TVB-N and TVC values were 3.84 mg/100g and 2.95 log_10_ CFU/g, respectively. As the storage time increased, a decrease in pH (6.85) occurred on the first day, and then gradually increased to 6.26 on the 7th day. This may be due to the production of lactic acid from the anaerobic digestion of glycogen and phosphate production from adenosine triphosphate degradation in pork [50]. However, microbial reproduction produces and releases a large number of volatile alkaline substances, resulting in a slow increase in pH once again. On the 6th day, the TVB-N and TVC values increased from 3.84 mg/100 g and 2.95 log_10_ CFU/g to 18.71 mg/100 g and 6.08 log_10_ CFU/g, respectively, representing an increase of 510.7% and 137.9%. These exceed the limits of 15 mg/100 g (GB5009.228-2016) and 6 log_10_ CFU/g (GB 4789.2-2016) according to Chinese standards, thus indicating that pork was not edible after 6 days of storage in the refrigerator [51].

#### 3.9.2. Color Variations of the Films

**Figure 10:**
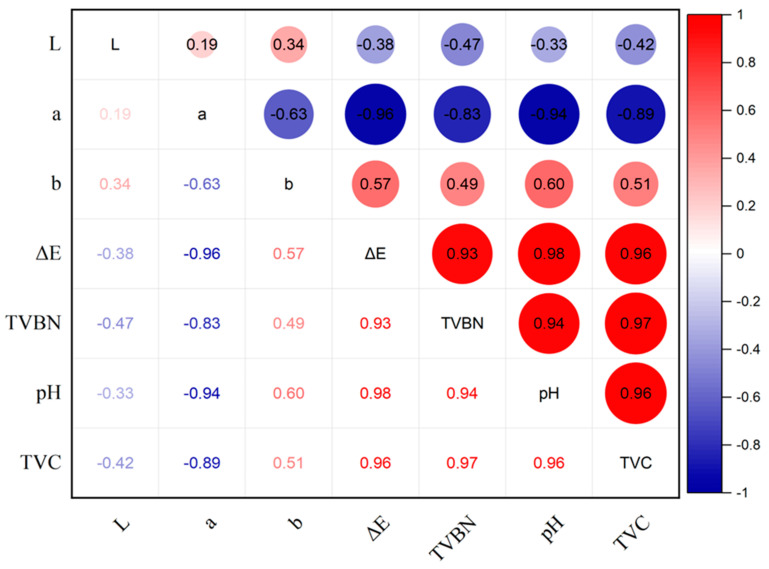
Correlation analysis between film color and quality parameters of pork.

As shown in Table 2, the freshness of pork changed with increasing storage time. The color of the film shifted from orange to light purple and finally to dark purple. The α-hydroxy anthraquinone in the cochineal molecules ionizes due to the alkaline nitrogenous compounds produced by the deterioration of pork, thus showing color variations from orange-red to purple [33]. On day 6, when the pork became inedible, only the PVA/GG-4 film exhibited visually distinguishable color changes that matched the variations in pork freshness. On account of the low content of cochineal, the color changes of the PVA/GG-2 film cannot indicate the freshness of pork in real time. In pork freshness monitoring applications, the PVA/GG-6 film exhibited a discernible color variation to the naked eye, but this color change occurred before pork spoilage. This may be due to the high content of cochineal in the film, which resulted in a more intense color change. The color variation of the pH-responsive film in pork freshness monitoring differs from that observed in the solution. This is due to the generation of various volatile alkaline gases (such as ammonia, trimethylamine, dimethylamine, methylamine, etc.) during pork spoilage. Moreover, the relative humidity of the pH-responsive film used in freshness monitoring is significantly lower than that of different pH solutions, hence resulting in a distinct color change compared to different pH buffers. In Figure 10, the ΔE* value of the PVA/GG-6 film showed a positive correlation with TVB-N, pH and TVC values with high correlation coefficients. The TVB-N value showed a positive correlation with both pH and TVC values, with a correlation coefficient ranging from 0.81 to 0.98. The results showed that the novel freshness indicator film was feasible for freshness monitoring of refrigerated pork. Consequently, both the application object and the pH-colorant content strongly influence the effectiveness of freshness monitoring using this indicator film.

## 4. Conclusions

As the pH increased from 2 to 12, the cochineal color shifted from orange to red and then to purple. The FIIR spectrum of the pH-responsive films indicated successful immobilization of CSN in the PVA/GG matrix, which can be attributed to newly generated interactions between the polymer matrix and the natural dye. The wettability and swelling properties of the pH-responsive films were influenced by the incorporation of CSN. Due to the poor photostability of cochineal, the photostability of pH-responsive films was significantly enhanced by encapsulation of cochineal with amylopectin particles. The release of cochineal from pH-responsive film followed the Fickian diffusion mechanism, which can be described by both a first-order model and a second-order model. The excellent antioxidant and antibacterial activities of pH-responsive films resulted from the release of cochineal. The ΔE* value of the PVA/GG-6 film showed a positive correlation with the TVB-N and TVC values of pork, with a high correlation coefficient. The color change of the PVA/GG-6 film can fully indicate the spoilage of refrigerated pork, enabling consumers to judge the freshness of cold meat products with their naked eyes.

## Figures and Tables

**Table 1:** Physical properties of the films.

Physical Properties	Cochineal	PVA/GG Film	PVA/GG-2 Film	PVA/GG-4 Film	PVA/GG-6 Film	PVA/GG-8 Film
*L*	17.33 ± 1.63 ^d^	66.17 ± 0.75 ^a^	59.17 ± 0.75 ^b^	57.83 ± 4.07 ^c^	56.00 ± 1.09 ^c^	51.50 ± 1.22 ^c^
*a*	13.00 ± 0.63 ^c^	−0.50 ± 0.55 ^e^	7.67 ± 0.52 ^d^	21.00 ± 1.26 ^b^	21.50 ± 0.84 ^b^	28.83 ± 1.17 ^a^
*b*	3.00 ± 1.27 ^f^	7.50 ± 0.55 ^e^	10.50 ± 1.22 ^d^	23.17 ± 0.75 ^b^	25.00 ± 0.89 ^b^	29.67 ± 1.51 ^a^
ΔE*	75.48 ± 1.69 ^a^	7.89 ± 0.55 ^e^	13.29 ± 1.01 ^d^	31.38 ± 1.02 ^c^	33.37 ± 2.12 ^c^	42.43 ± 1.89 ^b^
SI (%)	\	372.32 ± 23.58 ^a^	326.67 ± 19.54 ^c^	366.12 ± 29.18 ^a^	365.24 ± 22.26 ^a^	361.43 ± 28.84 ^b^
WS (%)	\	38.76 ± 1.57 ^a^	36.84 ± 1.93 ^b^	36.51 ± 1.81 ^b^	36.07 ± 2.18 ^b^	36.43 ± 2.32 ^b^
WVP (×10^−11^ g m^−1^ s^−1^ Pa^−1^*)*	\	13.27 ± 0.46 ^a^	12.83 ± 0.32 ^b^	12.67 ± 0.27 ^b^	12.46 ± 0.13 ^b^	12.39 ± 0.23 ^b^
WCA (°)	\	59.72 ± 1.62 ^a^	58.33 ± 1.32 ^b^	57.96 ± 1.03 ^b^	57.81 ± 1.01 ^b^	57.39 ± 1.23 ^b^

Values are displayed as mean ± SD (n = 5). Different letters in the same column present significant differences (*p* < 0.05).

**Table 2:** The change of quality parameters (TVB-N, pH, TVC values) in the pork as well as the color variation of pH-responsive films at different storage times.

Time (D)	pH	TVB-N (mg/100 g)	TVC (log_10_ CFU/g)	Color Variations of the pH-Responsive Films (4 °C)		
				PVA/GG-2	PVA/GG-4	PVA/GG-6
0	5.11 ± 0.09 ^e^	3.84 ± 0.08 ^h^	2.95 ± 0.21 ^h^	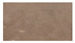	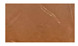	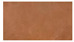
1	5.48 ± 0.14 ^d^	5.80 ± 0.09 ^g^	3.41 ± 0.33 ^g^	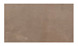	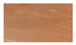	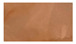
2	5.82 ± 0.09 ^b^	7.03 ± 0.18 ^f^	3.88 ± 0.15 ^f^	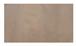	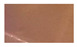	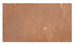
3	5.64 ± 0.15 ^c^	8.78 ± 0.19 ^e^	4.32 ± 0.23 ^e^	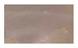	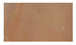	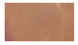
4	6.01 ± 0.05 ^b^	10.86 ± 0.31 ^d^	4.82 ± 0.21 ^d^	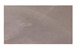	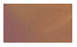	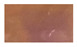
5	6.15 ± 0.06 ^a^	12.75 ± 0.39 ^c^	5.07 ± 0.19 ^c^	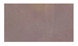	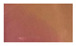	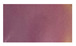
6	6.20 ± 0.13 ^a^	18.71 ± 0.58 ^b^	6.08 ± 0.22 ^b^	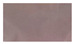	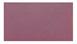	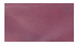
7	6.26 ± 0.12 ^a^	23.45 ± 0.42 ^a^	7.02 ± 0.17 ^a^	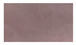	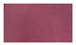	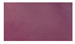

Values are displayed as mean ± SD (n = 3). Different letters in the same column present significant differences (*p* < 0.05).

## Data Availability

All related data and methods are presented in this paper. Additional inquiries should be addressed to the corresponding author.

## Supplementary Materials

The following supporting information can be downloaded at: https://www.mdpi.com/article/10.3390/foods12122316/s1, Figure S1: The standard curve of Cochineal; Figure S2: Color stability of PVA/GG-2, PVA/GG-4, PVA/GG-6, PVA/GG-8 films and PVA/GG-2CL, PVA/GG-4CL, PVA/GG-6CL, PVA/GG-8CL films (25 °C); Figure S3: Bacterial growth curves (A,C), corresponding antibacterial efficiency (B,D).
