# Supplementary material for: Preparation of pH-Responsive Films from Polyvinyl Alcohol/Agar Containing Cochineal for Monitoring the Freshness of Pork
Source: Foods. 2023 Jun 8;12(12):2316. doi: 10.3390/foods12122316 (PMC10297249; doi:10.3390/foods12122316)
Supplement: Supplementary file 1 [file foods-12-02316-s001.zip › foods-2410632-supplementary.pdf]

Preparation of pH-Responsive Films from Polyvinyl Alcohol/Agar Containing Cochineal for Monitoring the Freshness of Pork

Danfei Liu †, Yunfei Zhong †, Yumei Pu, Xiaoxuan Li, Siyuan Chen and Changfan Zhang *

School of Packaging and Materials Engineering, Hunan University of Technology, Zhuzhou 412007, China; m18080502003@stu.hut.edu.cn (D.L.); yfzhong@hut.edu.cn (Y.Z.); m21085600005@stu.hut.edu.cn (Y.P.); m22077300009@stu.hut.edu.cn (X.L.); m20085600011@stu.hut.edu.cn (S.C.)

* Correspondence: zcf@hut.edu.cn; Tel.: +86-0731-22182180

† These authors contributed equally to this work.

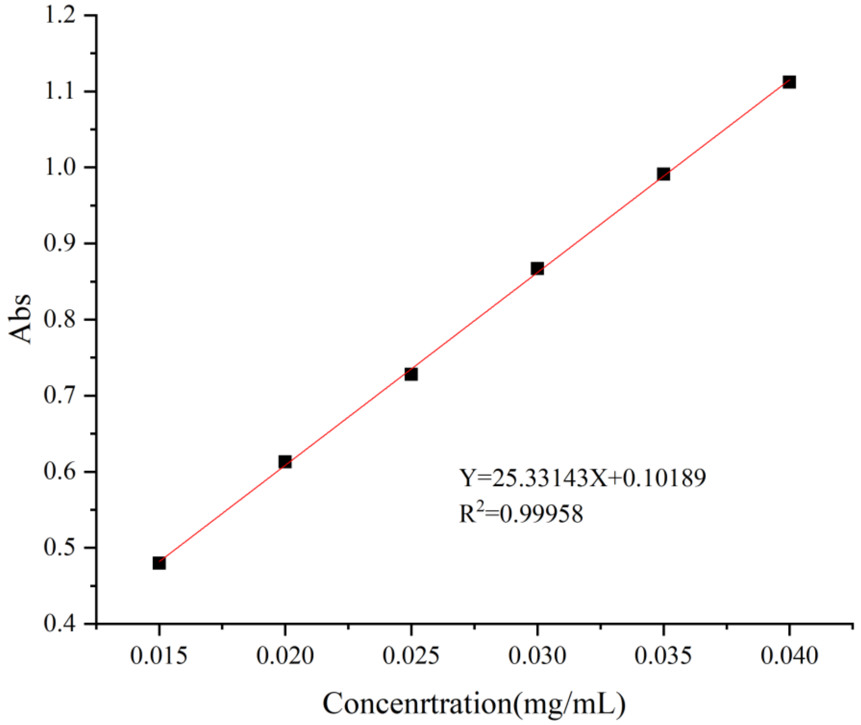

Figure S1. The standard curve of Cochineal.

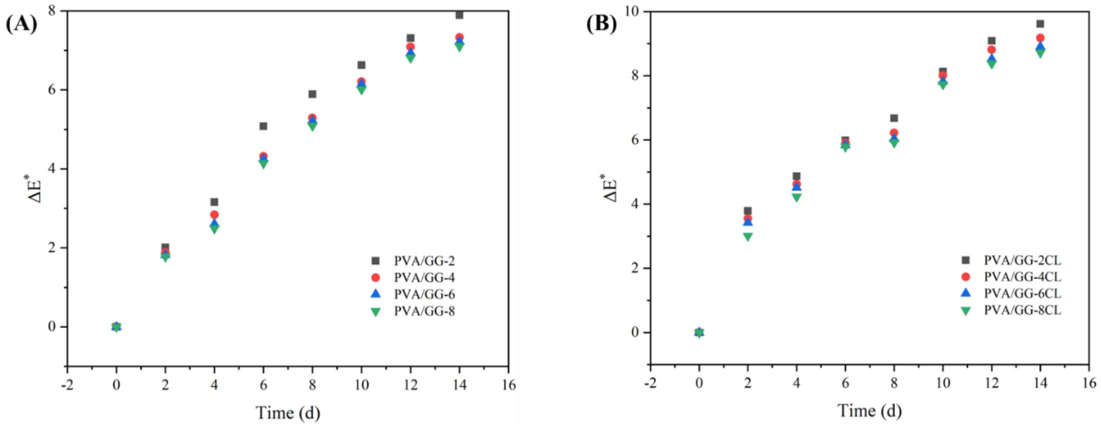

Figure S2. Color stability of PVA/GG-2, PVA/GG-4, PVA/GG-6, PVA/GG-8 films and PVA/GG-2CL, PVA/GG-4CL, PVA/GG-6CL, PVA/GG-8CL films (25 °C).

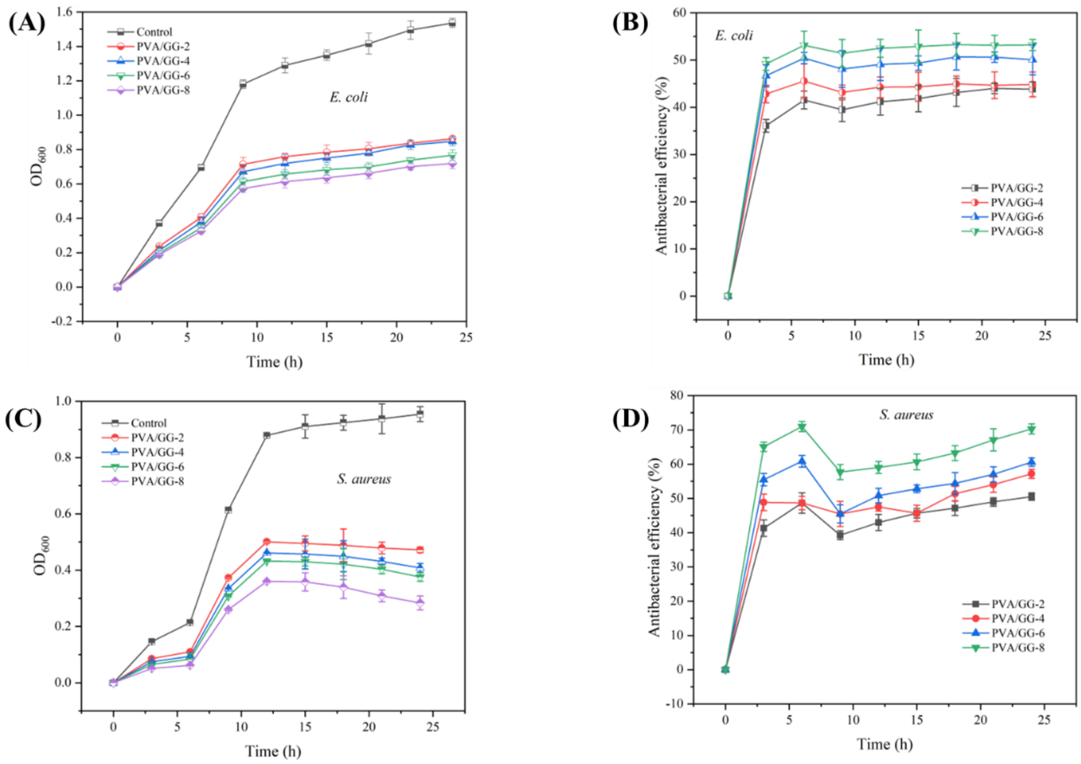

Figure S3. Bacterial growth curves (A, C), corresponding antibacterial efficiency (B, D).
